# Body Image of Highly Trained Female Athletes Engaged in Different Types of Sport

**DOI:** 10.1155/2018/6835751

**Published:** 2018-01-31

**Authors:** Adam Kantanista, Agata Glapa, Adrianna Banio, Wiesław Firek, Anna Ingarden, Ewa Malchrowicz-Mośko, Paweł Markiewicz, Katarzyna Płoszaj, Mateusz Ingarden, Zuzanna Maćkowiak

**Affiliations:** ^1^Department of Didactics of Physical Activity, Poznan University of Physical Education, Królowej Jadwigi 27/39, 61-871 Poznań, Poland; ^2^Youth Council of the Polish Olympic Academy, Wybrzeże Gdyńskie 4, 01-531 Warszawa, Poland; ^3^Physical Activity, Sport, and Recreation Focus Area, North-West University, Private Bag X600, Potchefstroom 2520, South Africa; ^4^Faculty of Physical Culture and Health Promotion, Szczecin University, Piastów 40B, 71-65 Szczecin, Poland; ^5^Department of Organization and History of Sport, Józef Piłsudski University of Physical Education in Warsaw, Marymoncka 34, 00-968 Warszawa, Poland; ^6^Department of Cultural Foundations of Tourism and Recreation, Poznan University of Physical Education, Królowej Jadwigi 27/39, 61-871 Poznań, Poland; ^7^Academic Sports Association, Maria Curie-Skłodowska University in Lublin, Plac Marii Curie-Skłodowskiej 5, 20-031 Lublin, Poland; ^8^Department of Pedagogy and Psychology, Józef Piłsudski University of Physical Education in Warsaw, Marymoncka 34, 00-968 Warszawa, Poland; ^9^Department of Physical Activity Study and Health Promotion, Poznan University of Physical Education, Królowej Jadwigi 27/39, 61-871 Poznań, Poland

## Abstract

**Background:**

The aim of the study was to evaluate differences in body image across different types of sports in highly trained female athletes.

**Methods:**

242 female individuals, aged 13–30 years (M = 20.0, SD = 4.5), representing aesthetic sports (*n* = 56) and nonaesthetic sports (*n* = 186), were recruited from different sports clubs in Poland. Body image, BMI, age, the level of competition attained, and the training background of participants were recorded.

**Results:**

One-way ANOVA showed differences in the body image of athletes engaged in different types of sport (*F*(11,230) = 4.10, *p* < 0.001, and *η*^2^ = 0.16). The model predicting the body image of female athletes was significant (*F*(5,236) = 10.40, *p* < 0.001); the adjusted *R*^2^ = 0.163. Type of sport explained 7.1% (*β* = –0.263, *p* < 0.001), age explained 4.5% (*β* = 0.341, *p* < 0.001), BMI explained 3.6% (*β* = –0.230, *p* < 0.001), and level of competition explained 0.9% (*β* = 0.153, *p* < 0.05) of variance in body image.

**Conclusions:**

The findings provide vital new knowledge which can be used by researchers and practitioners in designing educational programs on weight-related behaviors in female athletes. Such programs should be implemented especially in young female athletes participating in high-level sporting activities at an early stage.

## 1. **Introduction**

Female athletes experience both sociocultural and sport-specific pressure to change their weight, body, and appearance [1, 2] and they are at risk of developing dissatisfaction with their body. Body dissatisfaction involves negative thoughts and feelings about one's body and a perceived discrepancy between current and “ideal” body size [3]. The perception of a person's body may change in relation to the context in which he or she functions [4, 5]. Elite athletes reported as having both an athletic and a social body image [4–6]. Thus, body image can be measured in the context of either sport or daily life [7]. In the theoretical model proposed by Petrie and Greenleaf [8], body dissatisfaction is considered as a result of the internalization of societal and sport-specific pressures and can be a moderator of eating disorders among athletes. This sociocultural model is consistent with objectification theory, in which the human body is perceived as biologically and socioculturally developed. Women may internalize sociocultural beauty ideals [9, 10].

Among sport-specific pressures, the type of sport is a predictor of body dissatisfaction [11]. In a review of studies on body image in athletes and nonathletes, Varnes et al. [12] indicated that involvement in sport “protected” athletes from body image concerns, but this protection was less in women and in higher-level athletes. Similar results were recorded by Kong and Harris [13], in which female athletes from leanness sports (such as dancers and gymnasts) reported higher levels of body dissatisfaction than athletes engaged in nonleanness sports (e.g., ball sports), regardless of participation level. Moreover, elite athletes declared higher levels of body dissatisfaction than recreational and noncompetitive individuals [13]. In sports that emphasize aesthetic aspects, thinness, and appearance, body image disorders are more prevalent. Swami et al. [14] have observed that track athletes involved in recreational clubs (where leanness is actively promoted) had a higher degree of body dissatisfaction than martial artists and nonathletes. Ferrand et al. [15] indicated a higher level of dissatisfaction among synchronized swimmers than in the general population. Moreover, the athletes reported that they use different weight-loss methods (e.g., self-induced vomiting, fasting, and diuretics). Greater body dissatisfaction in dancers was also reported in a study by Robbeson et al. [16], whereas in the study of de Bruin et al. [17] female gymnasts (an aesthetic sport) reported the same perception of body shape and size when compared to nonelite gymnasts and nongymnasts. Based on interviews with female rugby players, cricketers, and netballers, Russell [5] found that participation in sport led to positive perceptions of one's body, but this effect was transient.

BMI may also be a factor related to body dissatisfaction. A positive correlation between body dissatisfaction and BMI has been observed in both general [18] and sport populations [19, 20]. For example, Karr et al. [20] found that higher BMI was associated with greater body dissatisfaction in female sport participants across different sport types (aesthetic/lean, nonaesthetic/lean, and nonaesthetic/nonlean).

Eating disorders are more often prevalent in elite female athletes who compete in aesthetic sports than those who participate in nonaesthetic sports [21]. That is why low satisfaction with body shape and physical appearance, along with a desire to be leaner, to improve sporting performance, is all indicative of eating disorders [22, 23]. Individuals dissatisfied with their body may undertake unhealthy behaviors (e.g., restrict dietary intake) or respond affectively (e.g., by experiencing negative emotions) and consequently this may contribute to the onset and persistence of an eating pathology [24].

In our study we focused on the self-perception of body image in highly trained female athletes, because, according to available literature, it seems that the phenomenon of body dissatisfaction in relation to different sports is still unexplored. Additionally, findings often vary by the type of sport, level of competition, age, and research methodology [20]. From a health perspective body satisfaction is one of the crucial factors in the prevention of unhealthy, weight-related behaviors, especially in female athletes. Additionally, body dissatisfaction can be used to predict eating disorders, especially among sportswomen [25], and these disorders may have very serious health consequences.

The aim of the study was to evaluate the level and differences in body image perceptions of highly trained female Polish athletes across different types of sports. A secondary aim was to investigate whether certain variables—participation in aesthetic and nonaesthetic sports, the level of competition, age, training background, and BMI—can be used to predict body image in female athletes.

Based on the findings of previous research, we hypothesized that perceived body image is more positive in female athletes representing nonaesthetic sports than in those engaged in aesthetic sports and that female athletes competing at national level have more positive body image than athletes competing at international level.

## 2. **Material and Methods**

### 2.1. Participants

This study involved a total of 242 individuals, aged 13–30 years (M = 20.0, SD = 4.5). Highly trained female athletes were recruited from different sports clubs in Poland, representing aesthetic sports, synchronized swimming (*n* = 19), gymnastics (*n* = 15), and dance sport (*n* = 22), and nonaesthetic sports, floorball (*n* = 33), soccer (*n* = 23), volleyball (*n* = 21), basketball (*n* = 19), karate (*n* = 21), swimming (*n* = 17), rugby (*n* = 20), field hockey (*n* = 21), and athletics (sprint, *n* = 11). Aesthetic sports were defined as sports in which athletes are judged with aesthetic components. In nonaesthetic sports such components are not judged. Weight and height were self-reported by the athletes. The data recorded were used to calculate BMI. Mean body height was 168.4 cm (SD = 10.4), mean body weight was 59.7 kg (SD = 12.6), and mean BMI was 20.9 kg/m^2^ (SD = 3.9). Only athletes competing at national or international level were included in the study. Among participants with a competitive sporting career, which ranged in length from 0.5 to 21 years (M = 9.0, SD = 4.7), 29.3% competed at national and 70.7% at international level. The research was conducted according to the principles of the Declaration of Helsinki. All participants took part in the study voluntarily and were informed that they could discontinue their involvement at any time.

### 2.2. Body Image Measure

Body image was assessed using the Feelings and Attitudes towards Body Scale incorporated in the Body Investment Scale developed by Orbach and Mikulincer [26]. The scale comprises six statements (e.g.,* I am satisfied with my appearance* and* I feel comfortable with my body*). Participants scored each statement on a 5-point scale ranging from* Absolutely disagree *to* Absolutely agree *(corresponding to the point values 0–4, resp.). The global integrated score therefore ranged from 0 to 24 points. The higher the cumulative score is, the more positive the athlete was about her body image. We decided to use the Feelings and Attitudes towards the Body Scale because it was adapted to Polish population. The original scale was translated into the national language (Polish) and then translated back into English for confirmation by the Health Behavior in School-aged Children project's International Coordinating Centre [27, 28]. This scale was used in research on body image in adolescents and adults [27, 29, 30]. In our study, the scale's internal consistency, established using Cronbach's alpha test, was 0.90.

### 2.3. Statistical Analysis

Descriptive statistics and *t*-tests were used to examine differences in the variables recorded for the athletes representing both aesthetic and nonaesthetic sports. To compare the percentage of participants competing at different levels, a test to establish different proportions was used. A one-way ANOVA compared the body image of the athletes from the different types of sports. Tukey's Honestly Significant Difference (HSD) post hoc test was employed to conduct multiple detailed comparisons. Eta squared (*η*^2^) was calculated to determine the percentage of variance explained by a particular effect. Stepwise forward selection regression analyses were conducted in order to investigate whether a statistically significant proportion of variance in body image was explained by participation in aesthetic and nonaesthetic sports, the level of competition, BMI, and age. For all statistical analyses, the level of significance was set at *p* ≤ 0.05. Statistical analyses were carried out using STATISTICA 10 (StatSoft, Inc.).

## 3. **Results**

Table 1 presents the main characteristics of the athletes participating in the different types of sports. The athletes from aesthetic sports were younger (*p* < 0.001), of lower height (*p* < 0.001), and of lower BMI (*p* < 0.01) and declared a more positive body image (*p* < 0.05) than the athletes from nonaesthetic sports.

**Figure 1:**
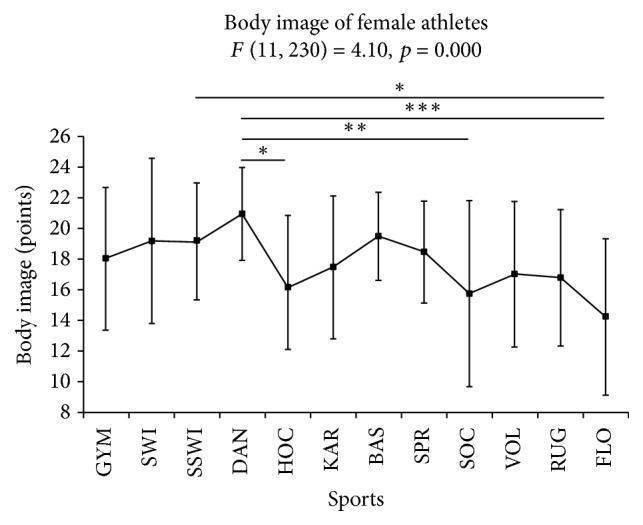
Body image perception of female athletes from different sport disciplines: GYM, gymnastics; SWI, swimming; SSWI, synchronized swimming; DAN, dance sport; HOC, field hockey; KAR, karate; BAS, basketball; SPR, athletics (sprint); SOC, soccer; VOL, volleyball; RUG, rugby; FLO, floorball. ^*∗*^*p* ≤ 0.05, ^*∗∗*^*p* ≤ 0.01, and ^*∗∗∗*^*p* ≤ 0.001.

One-way ANOVA showed differences in the body image of the athletes engaged in different types of sport (*F*(11, 230) = 4.10, *p* < 0.001, and *η*^2^ = 0.16). The HSD post hoc tests indicated differences in body image between dancers and field hockey players (*p* < 0.05), soccer players (*p* < 0.01), and floorball players (*p* < 0.001) and between floorball players and synchronized swimmers (*p* < 0.05) (Figure 1).

In the model predicting the body image of the athletes (Table 2), five variables [sport type (aesthetic/nonaesthetic), level of competition, age, training background, and BMI] were included. The model was significant (*F*(5, 236) = 10.40, *p* < 0.001), and the adjusted *R*^2^ = 0.163. Among significant variables, the type of sport explained 7.1% (*β* = –0.263, *p* < 0.001), age explained 4.5% (*β* = 0.341, *p* < 0.001), BMI explained 3.6% (*β* = –0.230, *p* < 0.001), and level of competition explained 0.9% (*β* = 0.153, *p* < 0.05) of variance in body image. A significant positive effect of age and level of competition was obtained, which indicates that body image perception was more positive as the age of the athletes increased and in athletes competing at an international level compared to those competed at a national level. We observed a significant negative effect of type of sport and BMI, which indicates a more negative image in athletes engaged in nonaesthetic sport compared to aesthetic sports. Furthermore, body image was more negative in athletes with a higher BMI.

## 4. **Discussion**

In this study, we evaluated the level and differences in the body image among elite female athletes from different types of sport. We found that athletes who represented aesthetic sports declared a more positive body image than those representing nonaesthetic sports. In the model we created to predict body image among these athletes, we found that the type of sport (aesthetic/nonaesthetic) as well as age, BMI, and level of competition was all significant. The results emphasize the importance of these factors in the context of body image.

Our findings did not confirm our hypothesis and indeed contrast with previous research, which indicated that women participating in aesthetic sports experience greater body dissatisfaction than those in other sports [13, 14]. For example, Kong and Harris [13] have observed greater general and sport-related body dissatisfaction in leanness-focused female athletes compared to athletes engaged in nonleanness sports. Rose [31] has indicated that athletes from appearance-focused sports like gymnastics and tennis showed less-positive body esteem and greater concern about weight than athletes in other disciplines, for which appearance did not matter. However, Krentz and Warschburger [23] found that athletes from aesthetic sports did not differ from recreationally active athletes with respect to general body dissatisfaction. Moreover, Torstveit et al. [21] have shown that fewer athletes competing in leanness sports than athletes competing in nonleanness sports were dissatisfied with their body.

The explanation of our results may relate to the fact that our study group of athletes from aesthetic sports had a lower BMI than those from nonaesthetic sports. BMI was a significant factor explaining body image in these sportswomen. According to Karr et al. [20], greater body dissatisfaction among participants in high-school sports was associated with higher BMI. In the study of Swami et al. [14], BMI was a stronger predictor of body dissatisfaction than sport type. The slim and fit body is an idealized female body form in Western societies [32, 33], which may be promoted via friends, family, and the media [34]. As a result, athletes from aesthetic sports might be more satisfied with their body within their social environment.

In our study, the level of competition was a significant predictor of body image; those athletes who competed internationally had a more positive perception of their body image than those who performed nationally. These findings contrast with the results of a meta-analysis, in which elite athletes (those competing at national or international levels), in comparison with sportswomen at other levels, were found to show the highest degree of risk for body image disturbance [35] and experienced more dissatisfaction with their body [36]. However, our investigation involved a relatively homogeneous group. Competition nationally and internationally is characterized by a similar determination of players to win. Nevertheless, competing at an international level may be associated with more experience and greater awareness of their body and thus a more positive body image among athletes. Moreover, the participants in the present study were between 13 and 30 years of age. A similar age range of female athletes has been analyzed in other crucial studies on body image and related topics [7, 13, 21, 37]. We observed that body image perception was more negative in younger athletes. This may be a consequence of the puberty period in which body fat percentage increases and breast and hips develop [38]. Consequently, young athletes who compete at an elite level might perceive their body more negatively. On the other hand, older athletes are more experienced in highly competitive sports environments and may also have greater athletic self-efficacy, which is associated with lower body dissatisfaction [20].

It is also possible that in our study the more positive body image perception in women involved in aesthetic sports was related to other factors. The results might be confounded by competition level, age, and BMI or there might be interaction among these variables. Body image perception in females may be the result of a combination of factors that incorporate both interpersonal and intrapersonal factors [3]. Additionally, pressure from the coaches, for example, by making negative weight-related comments, was not evaluated. Such comments can upset athletes and make them become more focused on their bodies [39]. In the study by Kong and Harris [13], more than 60% of elite female athletes reported pressure from coaches concerning body shape. Similar results have been obtained by other authors [40, 41]. Coaches are thus powerful social agents in athletes' body image perceptions [4]. In the model proposed by Petrie and Greenleaf [8] and according to Reel et al. [1], parents, friends, judges, media, competitiveness, and training regime may all also add pressure on female athletes and promote body dissatisfaction.

In the present study, we recorded a statistically significant more positive perception of body image in dancers than in field hockey players, soccer players, and floorball players. Likewise, synchronized swimmers had a more positive body image than floorball players. This result is in contrast with the results of other studies. Robbeson et al. [16] observed that more female dancers than controls (nonathletes) were dissatisfied with their body. Furthermore, the discrepancy between actual and desired body weight was greater for dancers than in the control group. Hincapié and Cassidy [42] have found that body dissatisfaction is linked to dance, a discipline in which leanness is required. According to Ferrand et al. [43], synchronized swimmers reported greater negative feelings about their appearance than athletes representing team sports and nonathletes. A more negative perception of body image by female athletes pursuing team sports, compared to their counterparts in aesthetic sports (dancers and synchronized swimmers), may be related to the characteristics of these disciplines. Field hockey, soccer, and floorball are invasive games in which the players can compete in direct contact. These games are perceived in whole or in part as “masculine” [12]. In relation to the Petrie and Greenleaf [8] model, athletes are strongly exposed to sport and sociocultural pressure regarding weight and body shape. In many sports (e.g., synchronized swimming and dance), low body fat and thinness are crucial variables for performance [44]. These factors are also promoted by the media as important for an attractive appearance and ideal body shape [45]. From a sociocultural point of view, therefore, the more positive body image of dancers and synchronized swimmers is reasonable. De Bruin et al. [7] suggest that in a social environment athletes may be satisfied with their body, but in a sporting context, they may be dissatisfied with it.

Athletes are a subpopulation which is particularly prone to body image distortion and eating disorders. Stice and Shaw [46] indicated in their study that, in turn, heightened dissatisfaction with the body increases the risk of various adverse outcomes, for example, eating disorders. According to Stice [47], body dissatisfaction may be viewed as a primary precursor of eating disorders. Abnormal eating, which includes restrictive eating, overeating, skipping meals, and binge-eating and the use of diet pills and diuretics [48] are undertaken by athletes to achieve desired weight or body shape. Therefore, there is a need for an early investigation of potential correlates of eating disorders, including body image, to protect health of female athletes.

Our study has some limitations but also a number of strengths. One key strength is the number of highly trained Polish female athletes investigated. Furthermore, this is the first research on body image conducted among highly trained female athletes in Poland. These results should, therefore, provide a useful basis for future research. A limitation is the self-reporting nature of the survey and the use of self-reported weight and height to calculate BMI. It should be noted that in our study only highly trained sportswomen were examined. In other studies, the body image of athletes and nonathletes were compared. This difference should be kept in mind when analyzing the results of the present study.

## 5. **Conclusions**

This study presents an initial assessment of the differences in body image perception across different types of sport held by highly trained Polish female athletes. We found that those from aesthetic sports declared a more positive body image than athletes engaged in nonaesthetic disciplines; moreover, the type of sport, age, BMI, and level of competition play a significant role in how body image is perceived. Our findings contrast with previous research on body image in females competing in aesthetic and nonaesthetic sports. The findings thus provide vital new knowledge which can be used by researchers and practitioners in designing educational programs on weight-related behaviors in female athletes. Such programs should be addressed to both athletes and coaches because, according to Vaughan et al. [49], there is a lack of knowledge and confidence in identifying the early symptoms (e.g., body dissatisfaction) of eating problems among athletes. Because of greater body dissatisfaction in young athletes, interventions that improve body image perception should be implemented in female adolescent cohorts participating in high-level sporting activities at an early stage.

## Figures and Tables

**Table 1:** Characteristics of female athletes representing different types of sport and differences between aesthetic and nonaesthetic sports.

	Age (years)	Weight (kg)	Height (cm)	BMI (kg/m^2^)	Training background (years)	Level of competition^*∗*^		Body image (points)
						1	2	
	M ± SD					%		M ± SD
Aesthetic sports								
Total	17.6 ± 5.2	47.3 ± 12.0	160.3 ± 12.2	18.1 ± 2.7	8.2 ± 4.8	16.1	83.9	19.5 ± 3.9
Gymnastics	13.3 ± 0.5	31.9 ± 4.3	145.2 ± 6.5	15.1 ± 1.5	5.1 ± 1.0	40.0	60.0	18.0 ± 4.7
Synchronized swimming	14.5 ± 2.4	48.0 ± 8.5	161.7 ± 9.3	18.3 ± 2.3	4.9 ± 2.6	10.0	90.0	19.2 ± 3.8
Dance sport	23.2 ± 3.1	57.0 ± 5.7	169.5 ± 5.9	19.9 ± 1.8	13.2 ± 3.2	4.5	95.5	20.9 ± 3.0

Nonaesthetic sports								
Total	21.7 ± 3.8	63.4 ± 10.8	170.8 ± 8.3	21.7 ± 3.8	9.2 ± 4.7	33.3	66.7	16.8 ± 4.9
Floorball	21.1 ± 2.6	64.7 ± 17.5	167.2 ± 7.1	23.4 ± 7.8	8.9 ± 3.3	39.4	60.6	14.2 ± 5.1
Soccer	20.4 ± 4.1	60.2 ± 5.7	168.3 ± 5.0	21.2 ± 1.6	10.2 ± 4.0	8.7	91.3	15.7 ± 6.1
Volleyball	17.2 ± 0.8	67.6 ± 6.8	178.9 ± 6.3	21.1 ± 1.3	6.1 ± 1.7	0.0	100.0	17.0 ± 4.7
Basketball	22.9 ± 3.9	64.6 ± 9.8	176.9 ± 9.8	20.6 ± 1.8	10.2 ± 4.1	63.2	36.8	19.5 ± 2.9
Karate	24.4 ± 3.2	58.5 ± 7.7	166.7 ± 7.0	21.0 ± 1.9	10.7 ± 4.6	33.3	66.7	17.5 ± 4.6
Swimming	22.4 ± 4.1	67.8 ± 11.6	178.7 ± 5.2	21.1 ± 2.7	14.1 ± 4.7	11.8	88.2	19.2 ± 5.4
Field hockey	21.8 ± 4.1	62.2 ± 7.8	166.9 ± 7.9	22.3 ± 1.9	10.9 ± 4.1	9.5	90.5	16.1 ± 4.0
Athletics	24.5 ± 2.5	59.1 ± 3.8	168.7 ± 4.6	20.8 ± 1.0	10.5 ± 3.1	18.2	81.8	18.5 ± 3.3
Rugby	23.4 ± 3.8	64.4 ± 9.8	168.5 ± 6.3	22.6 ± 2.5	2.6 ± 1.6	90.0	10.0	16.8 ± 4.4

*p*	<0.001	0.299	<0.001	<0.01	0.812	0.781	0.091	<0.05

^*∗*^Level of competition: 1: national; 2: international.

**Table 2:** Stepwise forward selection regression analysis for variables predicting body image (*N* = 242).

Steps	Variable	*B*	SE* B*	*β*	SE *β*	*T*	*p*
1	Sport type (aesthetic/nonaesthetic)	−2.757	0.711	−0.263	0.068	−3.87	<0.001
2	Age	0.363	0.088	0.341	0.083	4.10	<0.001
3	BMI	−0.285	0.080	−0.230	0.064	−3.55	<0.001
4	Level of competition	1.613	0.721	0.153	0.068	2.23	<0.05
5	Training background	−0.104	0.087	−0.101	0.084	−1.20	0.231

*Note*. *R*^2^ = 0.071 in step 1, Δ*R*^2^ = 0.045 in step 2, Δ*R*^2^ = 0.036 in step 3, Δ*R*^2^ = 0.009 in step 4, and Δ*R*^2^ = 0.002 in step 5; sport type coded 0 = aesthetics and 1 = nonaesthetics; level of competition coded 0 = national and 1 = international.
